# Plant-based snacking: research and practical applications of pistachios for health benefits

**DOI:** 10.1017/jns.2021.77

**Published:** 2021-10-01

**Authors:** Jennette Higgs, Kathryn Styles, Arianna Carughi, Michael A. Roussell, France Bellisle, Wiebke Elsner, Zhaoping Li

**Affiliations:** 1 Food to Fit Ltd, London, UK; 2 American Pistachio Growers, Fresno, CA 93720, USA; 3 Janus Nutrition LLC, New York, NY, USA; 4 NutriPsy Consult, Paris, France; 5 Berufsakademie Nord, University of cooperative Education, Hamburg, Germany; 6 Center for Human Nutrition, David Geffen School of Medicine, University of California, Los Angeles, CA, USA

**Keywords:** Appetite, Gestational diabetes, Nutrients, Pistachio nuts, Plant-based snacking, BMI, body mass index, CI, confidence interval, CVD, cardiovascular disease, GDM, gestational diabetes mellitus, GIGT, gestational impaired glucose tolerance, GIP, gastric inhibitory polypeptide, WWB, whole wheat bread

## Abstract

Pistachio nuts are a nutrient-dense source of good quality plant protein, commonly consumed as a minimally processed snack food or ingredient. The present paper is based on a symposium held during the 13th FENS (Federation of European Nutrition Societies) 2019 conference in Dublin that explored recent research and practical applications of pistachios as a plant-based snack, in particular, for appetite control and healthy weight management; and for glycaemic control during pregnancy. Individual nut types, whilst similar in nutritional composition, have unique characteristics which may have a significant impact on potential health effects. Recognising this, the further purpose here is to explore future research needs for pistachios, based on work completed to date and the discussion that ensued among researchers at this event, in order to advance the full scope of health benefits from pistachios, in particular, taking into account of both sustainability and nutritional health.

## Introduction

Research on the health benefits of nuts is now extensive and supports their regular inclusion in the diet^(1–6)^. Whilst the collective results from nut studies can provide a general consensus overview of the nutrition and health benefits from their regular inclusion in the diet, it cannot be assumed that findings from mixed nuts studies are applicable to all individual nut types. This consideration is a fundamental criterion characterising the EU nutrition and health claims regulations (EUNHCR)^(7)^ and as such, it remains important to conduct research on individual nuts to establish their effectiveness in contributing to reducing risk of chronic diseases, such as CVD, obesity, metabolic diseases and cancers.

The present paper focuses on topics relevant to the FENS 2019 theme (*Malnutrition in an obese world*), which were presented as new research findings and subsequently published^(8,9)^. The aim here is to broaden awareness of these recent and novel pistachio research topics, respectively: satiety effects and healthy weight management; and glycaemic effects during pregnancy. Both are relevant to the potential role of pistachios as a plant-based snack and hence the further inclusion of the broad nutrition and health attributes of pistachios provides valuable perspective to this narrative. It is recognised, however, that the paper does not provide a comprehensive research review of pistachios, rather it utilises as its basis the research provided during the FENS 2019 pistachio symposium, with additional research cited where the authors agreed that more detail was necessary to better explore a topic (Tables 1–3). A primary component of advancing further research on pistachios should include thorough systematic reviews of each topic.

**Table 1.** Observational studies and meta-studies investigating the association between nut intake and risk of adiposity/weight gain

Author reference	Design/Location	Variables	Key results
Guarneiri & Cooper^(52)^	Systematic review and meta-analysis of RCTs	Nut intake with or without dietary substitution AND body weight, BMI, waist circumference and % body fat	No significant change in body weight: SMD: 0⋅01 kg; 95 % CI: −0⋅07, 0⋅08 (without dietary substitution) and SMD −0⋅01 kg; 95 % CI: −0⋅11, 0⋅09 (with dietary substitution). No change in BMI or waist circumference for either type of study. Slight decrease in % body fat in those with dietary substitution: SMD: −0⋅32 %; 95 % CI: −0⋅61 %, −0⋅03 %
Xia *et al.*^(54)^	Systematic review and meta-analysis of RCTs	Pistachio nuts AND BMI, body weight and waist circumference	No significant associations with body weight and waist circumference; inverse association with BMI.
			BMI: WMD −0⋅18 kg/m^2^ (95 % CI: 0⋅26, −0⋅11 kg/m^2^). Body weight: −0⋅22 kg (95 % CI: −0⋅50, 0⋅07 kg). Waist circumference: WMD 0⋅76 cm (95 % CI: −0⋅11, 1⋅63 cm).
Liu *et al.*^(48)^	Prospective, longitudinal cohorts (NHS, NHS II, HPFS); USA	Change in nut intake AND weight change	Increasing daily nut intake associated with less long-term weight gain and lower risk of obesity. Weight gain >5 kg: RR: 0⋅77 (95 % CI: 0⋅73, 0⋅80) and a lower risk of becoming obese: RR 0⋅77 (CI: 0⋅74, 0⋅80).
Schlesinger *et al.*^(51)^	Systematic review and dose–response meta-analysis of prospective studies	Food group intake AND risk of overweight/obesity, abdominal obesity and weight gain	Inverse association for nuts and abdominal obesity. Abdominal obesity: RR: 0⋅42 (95 % CI: 0⋅31, 0⋅57).
Freisling *et al.*^(47)^	Prospective, longitudinal cohort (EPIC); Europe	Nut intake AND weight change	Inverse association for nut intake and weight gain and risk of obesity. Overweight/obesity: RR: 0⋅95 (95 % CI: 0⋅92, 0⋅98) fourth *v.* first quartile.
Li *et al.*^(3)^	Meta-analysis of:	Nut intake AND overweight/obesity	
	a) Prospective cohort studies b) RCTs		a) Inverse association with overweight and risk of obesity RR for every 1-serving/week increase in nut intake for overweight/obesity: 0⋅97 (95 % CI: 0⋅95, 0⋅98) for obesity: 0⋅95 (95 % CI: 0⋅89, 1⋅02). b) Inverse association with BMI, body weight and waist circumference. Body weight: WMD: −0⋅22 kg, (95 % CI: −0⋅40, −0⋅04), BMI: WMD: −0⋅16 kg/m^2^ (95 % CI: −0⋅31, −0⋅01) and waist circumference: WMD: −0⋅51 cm (95 % CI: −0⋅95, −0⋅07).
O'Neil *et al.*^(50)^	Cross-sectional; USA	Tree nut intake AND risk factors for CVD	Tree nut intake associated with lower BMI, waist circumference and a lower likelihood of overweight and obesity.
Flores-Mateo *et al.*^(24)^	Meta-analysis of RCTs	Nut-rich diets AND body weight, BMI and waist circumference	No significant effect including nuts on body weight, BMI or waist circumference in RCTs. Body weight: WMD: −0⋅47 kg (95 % CI: −1⋅17, 0⋅22 kg). BMI: WMD: −0⋅40 kg/m^2^ (95 % CI: −0⋅97, 0⋅17 kg/m^2^). Waist circumference: WMD: −1⋅25 cm (95 % CI: −2⋅82, 0⋅31 cm).
Mozaffarian *et al.*^(105)^	Prospective, longitudinal cohort (NHS, NHS II, HPFS); USA	Changes in life-style factor AND weight change	Inverse association for nuts and weight change: −0⋅78 (95 % CI: −1⋅31, −0⋅26).
Bes-Rastrollo *et al.*^(23)^	Prospective, longitudinal cohort (NHS); USA	Nut intake AND weight change	Nut intake (≥2 times/week compared with never/almost never) associated with a slightly lower risk of weight gain and obesity. Obesity: OR: 0⋅81 (95 % CI: 0⋅61, 1⋅08).
Bes-Rastrollo *et al.*^(106)^	Prospective, longitudinal cohort (SUN); Spain	Nut intake AND weight change	Nut intake (≥2 times/week compared with never/almost never) associated with a lower risk of weight gain. Overweight/obesity: OR: 0⋅73 (95 % CI: 0⋅48, 1⋅11) and weight gain: OR: 0⋅73 (95 % CI: 0⋅55, 0⋅96).
Schulz *et al.*^(107)^	Prospective, cohort (EPIC-Potsdam); Germany	Food groups AND weight change	Increased nut/seed intake decreased the likelihood of both large weight gain and loss. Weight gain: OR: 1⋅06 (95 % CI: 0⋅47, 2⋅39) for 100 g/d increase.

RCT, randomised controlled trial; BMI, body mass index; SMD, standard mean difference; CI, confidence interval; NHS, Nurses’ Health Study; NHS II, Nurses’ Health Study II; HPFS, Health Professionals Follow-Up Study; RR, relative risk; EPIC, European Investigation into Cancer and Nutrition; WMD, weighted mean difference; CVD, cardiovascular disease; OR, odds ratio; SUN, Seguimiento Universidad de Navarra.

**Table 2.** Randomised controlled trials evaluating the effect of pistachio intake on adiposity/weight gain

Author ref	Design/duration	Treatment/control	Result
Fantino *et al.*^(8)^	Parallel/ 12 weeks	44 g/d pistachios *v.* usual daily intake	No change in body weight or body composition.
Rock *et al.*^(29)^	Parallel/ 4 months	Group-based behavioural weight loss intervention with 42 g/d pistachios *v*. no pistachios	No change in body weight, BMI or waist circumference.
Carughi *et al.*^(58)^	Parallel/ 4 weeks	56 g/d pistachios *v*. isoenergetic savoury biscuit	No change in body weight.
Burns-Whitmore *et al.*^(59)^	Cross over/ 10 weeks	20 % energy from pistachios *v.* no pistachios	No significant differences in body weight or composition.
Gulati *et al.*^(56)^	Parallel/ 24 weeks	20 % of total energy from pistachios *v*. no pistachios. Pistachios substituted visible fat.	No change in body weight, lower waist circumference.
Hernández-Alonso *et al.*^(72)^	Cross-over	2 oz/d pistachios *v*. isoenergetic nut-free diet	No change in body weight, BMI or waist circumference.
Wang *et al.*^(55)^	Parallel/ 12 weeks	42 g, 70 g *v*. 0 g pistachios	No change in weight, BMI or waist circumference.
Li *et al.*^(57)^	Parallel/ 12 weeks	Weight loss intervention. Each diet included an isoenergetic snack of either 53 g of salted pistachios or 56 g of salted pretzels	Both groups lost weight. Pistachio group had a greater loss in BMI. Triacylglycerols were significantly lower in the pistachio group. No changes in cholesterol, insulin or glucose levels between groups.

BMI, body mass index.

**Table 3.** Studies looking at the effect of pistachios on gestational diabetes and pregnancy outcomes

Study	Design, intervention and endpoints	Results
San Carlos Gestational Diabetes Mellitus Prevention Study. Madrid, Spain	*Prospective, RCT- Parallel Intervention from 8th–12th GW until 24–28th GW. Mediterranean diet with ≥40 ml extra virgin olive oil and handful pistachios (25–30 g) daily (intervention); Control group followed a standard diet with limited fat intake.*	See 1–3 below.
1. Assaf-Balut *et al.*^(92)^	Primary endpoint: GDM incidence at 24–28th GW. Gestational weight gain, pregnancy-induced hypertension, caesarean section, preterm delivery, perineal trauma, small and large for gestational age and admissions to neonatal intensive care unit were also assessed.	Supplemented Mediterranean diet (intervention) had a lower incidence of GDM: 23⋅4 % (in the control group) compared to 17⋅1 %. Crude RR for GDM was 0⋅73 (95 % CI: 0⋅56–0⋅95) for intervention *v*. control. Intervention group had better maternal and neonatal outcomes (see Table 5).
2. Assaf-Balut *et al.*^(94)^	Prospective sub-analysis. Primary endpoint: composite of maternofoetal outcomes pregnancy with normoglycemia during pregnancy.	Risk of urinary tract infections, emergency caesarean sections, perineal trauma, large-for-gestational age and small-for-gestational age new-borns were significantly reduced in the intervention group. Women in this group had lower fasting glucose and HbA_1c_ levels at 24–28^th^ and 36–38^th^ GW than the control group.
3. Melero *et al.*^(93)^	Follow-up at 2-year post-partum. Primary endpoint: offspring health during first 2 years of life.	Shorter duration in hospital in intervention group (11⋅9 ± 25⋅2 days *v.* 6⋅8 ± 9⋅1 days). Significantly lower hospital admissions rate in intervention group due to bronchiolitis/asthma, antibiotic treatment and corticosteroid treatment in children born to women with pre-gestational BMI <25 kg/m^2^.
Feng *et al.*. Shanghai, China^(9)^	Randomised Controlled Cross-over Trial. Two isoenergetic test meals: 42 g pistachios or 100 g whole wheat bread. Acute effect of pistachios on glucose, insulin and gut-derived incretins in women with GDM or GIGT	Pistachio intake induced significantly lower post-prandial glucose, insulin and GIP levels but higher GLP-1 levels compared to whole wheat bread.

RCT, randomised control trial; GW, gestational weeks; GDM, gestational diabetes mellitus; RR, relative risk; CI, confidence interval; HbA1c, glycated haemoglobin; BMI, body mass index; GIGT, gestational impaired glucose tolerance; GIP, gastric inhibitory polypeptide; GLP-1, glucagon-like peptide-1.

## Nutritional value of pistachios

**Fig. 1.**
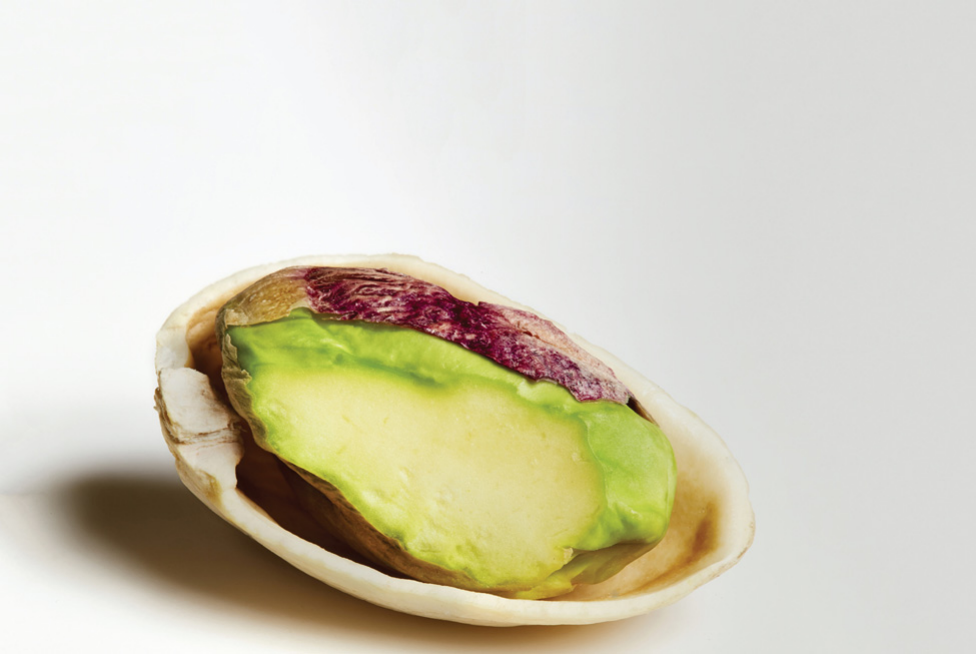
Pistachio nut (*Pistacia vera* L.) illustrating colours due to phytonutrient content, principally catechins, lutein, zeaxanthin, anthocyanins and chlorophyll.

Pistachios, as other nuts, are minimally processed nutrient- and energy-dense foods. Pistachios are a source of at least fifteen different micronutrients in significant amounts based on EU NHCR^(7)^ thresholds. As such, pistachios are high in copper, manganese, vitamin B6, thiamin, potassium, phosphorous, chromium and source of vitamin E and K, riboflavin, folate, magnesium, iron, zinc and selenium (Table 4), with smaller quantities of other micronutrients also present. They are also high in unsaturated and mono-unsaturated fats; contain linoleic acid (13.1 g/100 g), oleic acid (23⋅9 g/100 g) and plant sterols (210 mg β-sitosterol/100 g)^(10)^. Compared to other nuts^(11)^, pistachios have the lowest fat and highest potassium content and are amongst the highest in protein, fibre, vitamin K and phytosterol content. Pistachios also provide lutein and zeaxanthin (xanthophyll carotenoids) and phenolic compounds, including anthocyanins, flavonoids and proanthocyanidins, and their antioxidant capacity is considerable^(12)^. Their unique colour is due to the combination of these beneficial compounds^(13–15)^: the yellow colour from catechins, lutein and zeaxanthin; the green from chlorophyll; and the purple outer skin from anthocyanins (Fig. 1). According to the USDA, one serving of pistachios (28⋅35 g) contains forty-nine nuts^(10)^.

**Table 4.** Dry roasted pistachios nutrition composition^(10)^

	Nutrient content per 100 g
Energy (kJ/kcal)	2498/602
Fat (g)	46
– saturates (g)	5⋅6
– *mono-unsaturates* (g)	25
– polyunsaturates (g)	13
Carbohydrate (g)	17
– sugars (g)	7⋅7
*Fibre* (g)	10
Protein (g)	25
Salt (g)	0⋅015–1⋅1^a^
Vitamin E (mg)	2⋅2
Vitamin K (μg)	13
*Thiamin* (mg)	0⋅7
Riboflavin (mg)	0⋅23
*Vitamin B6* (mg)	1⋅1
Folic acid (μg)	51
*Potassium* (mg)	1010
*Phosphorus* (mg)	469
Magnesium (mg)	109
Iron (mg)	4
Zinc (mg)	2⋅3
*Copper* (mg)	1⋅3
*Manganese* (mg)	1⋅2
Selenium (μg)	10
*Chromium (*μg)	39^b^

RI, reference intake.

Source of claim possible (light shading); *High in* claim possible in the European Union (dark shading).

a Salt content variable according to distributor, e.g. ‘unsalted’, 0⋅015 g/100 g^(10)^; ‘lightly salted’ 0⋅938 g/100 g^(58)^ and salted 1⋅1 g/100 g^(10)^.

b Cabrera *et al.* ^(108)^.

The protein quality of pistachios has recently been evaluated using the DIAAS (digestible indispensable amino acid score) and PDCAAS (protein digestibility corrected amino acid score) methods. Raw and roasted pistachios were shown to have DIAAS values of 86 and 83 and PDCAAS of 73 and 81, respectively^(16)^. The discrepancy may be due to DIAAS values more accurately representing protein quality in heat-treated foods. Given these values, pistachios in both the raw and roasted forms are a ‘good’ quality protein^(17)^. A distinction is noteworthy here between protein quality, which considers the quantity of each of the essential amino acids needed to maintain adequate health and how easily digestible the protein is, *v*. ‘source of’ protein, which refers to the absolute protein content in a given food. Furthermore, regulatory definitions for ‘source of’ differ between countries, such that pistachios can be considered a ‘source of protein’ within the EU (≥12 % energy from protein)^(7)^ and yet are described as a ‘good source of protein’ in the USA (10–19 % of the daily value of protein per reference amount customarily consumed)^(18)^.

## Pistachios and health

The health benefits of nuts have been supported by a long history of epidemiological and clinical studies, with benefits being attributed to the healthy nutrient profile of nuts, particularly in the context of a Mediterranean diet, and their rich content of protective bioactive compounds^(6)^. Frequent nut consumption, including pistachio, has been associated with reduced risk of cardiovascular disease^(2)^, metabolic syndrome^(3)^ and all-cause mortality^(4)^ and beneficial effects on blood pressure^(19)^ (for comprehensive reviews, see Coates *et al.*^(20)^, Bitok *et al.*^(21)^ and Aune *et al.*^(22)^). Despite the fact that nuts, including pistachios, contain a significant amount of fat and are energy-dense foods, several epidemiological studies have provided strong evidence that nut consumption is associated with neither weight gain nor an increased risk of obesity^(23,24)^ (Table 1).

Growing clinical and epidemiological research supports the specific health benefits of pistachio consumption. They have been shown to improve diet quality^(25,26)^ and provide bioactive compounds with recognised properties for cardiovascular health^(27,28)^, weight management (Table 2) and glycaemic control^(9,11,29)^. Randomised controlled clinical studies have shown they lower CVD risk factors and oxidised low-density lipoproteins (LDL) cholesterol^(28)^. Systematic reviews and meta-analyses of RCTs suggest beneficial effects on blood pressure, endothelial function and on markers of glucose and insulin metabolism^(19,30)^. For comprehensive reviews of the health effects of pistachio nuts, see Bullo *et al.*^(9)^

### Plant-based diets

Plant-based diets, which emphasise plant foods but do not necessarily fully exclude animal foods^(31)^, have become centre stage both for nutritional health and climactic/sustainability reasons. An evidence-based focus on one of nature's unprocessed, plant food snacks is therefore timely. The EAT-Lancet recommendations emphasise a greater than 100 % increase in ‘healthy foods, such as nuts, fruits, vegetables and legumes’ by 2050 and a greater than 50 % decrease in ‘unhealthy foods such as red meat and sugar’, albeit with significant geographical differences, in order to move toward a global food system that is in tune with the stability of the Earth system as well as helping to reduce the global burden of non-communicable diseases^(32)^. Populations consuming or moving towards typical ‘western’ diets (with high fat/sugar/salt and insufficient fruits and vegetable intakes) are more likely to have higher obesity rates^(33)^, which then stimulate the drive for weight loss programmes within these societies. As a consequence, popular, restrictive fad diets have emerged^(34,35)^, promoted to achieve quick weight loss, which reduce food diversity, as well as quantity. Unlike health care professionally led weight loss programmes, they inevitably can result in micronutrients intakes below daily recommended amounts which could therefore result in long-term clinically relevant nutritional inadequacies^(34,36,37)^. Long-term dietary approaches for combatting obesity, whilst limiting energy intakes, must also accommodate adequate micronutrient needs and ideally fit with 21st-century sustainability goals. Tree nuts, such as pistachios, contribute to better nutrient adequacy and diet quality^(25,26)^, providing good protein quality^(16)^ and micronutrients. Malnutrition in an obese world provides a pertinent focus for exploring the potential usefulness of pistachios, as a plant-based snack option within effective weight management strategies that may also contribute to improved nutritional status and sustainability goals.

An aspect of sustainability that frequently arises in connection with agriculture is water usage, especially in semi-arid regions. Pistachio trees originated in the Middle East some 3–4000 years ago^(38)^. They are deciduous and thrive in dry climates and poor soil conditions^(39)^, each tree being productive for over 100 years^(40)^. A recent review of their efficiency of water use for cultivation indicates that yield remains satisfactory under drought conditions and is not significantly affected by moderate and properly timed, deficit-irrigation restrictions during the growing season^(39)^.

Regular nut intake is now recommended within national dietary guidelines around the world because of their nutrient contribution to the diet and reported health benefits. For example, in the USA, 5oz (approximately 150 g) of nuts are recommended weekly in combinations with seeds or soya products^(41)^; and Canada's Food guide recommends consuming plant-based sources of protein, including nuts, more regularly^(42)^. In France, the National Program for Nutrition and Health (PNNS) recommends the daily intake of a ‘small handful of unsalted nuts’ (a handful to reflect body size), highlighting their content in non-saturated lipids. Pistachios are among the explicitly recommended nuts^(43)^.

### Snacking

There is no consensus definition of ‘snacking’. In the scientific literature, snacking has been variously described as eating outside of culturally accepted ‘main’ meals; eating ‘non-core’ foods or foods with high fat, high energy and/or low micronutrient density; or eating in the absence of physiological hunger^(44)^. Depending on the definition, ‘snacking’ can exert beneficial or adverse effects. Evidence suggests that consuming regular snacks between main meals is one mechanism facilitating the adjustment of energy intake to energy requirements^(45)^. By contrast, impulsive snacking in response to external stimuli, particularly while watching TV or other screens, without paying attention to the act of eating and in the absence of hunger can impair appetite control and contribute to weight gain. Both nutrient quality of snack foods and circumstances of eating are important considerations determining the value of snacks in the diet. Nuts can therefore provide a suitable snack option due to their nutrient density.

## Regular intake of nuts and body weight

Due to their energy density, recommending nut consumption raises concerns in terms of weight management in the context of the worldwide epidemic of obesity and weight-concerned consumers may be reluctant to include nuts in their daily diet. Neale *et al.* ^(46)^ explore the various barriers that may account for the discrepancy between recommended and actual nut intakes^(46)^. Robust research, both epidemiological and randomised controlled clinical studies (RCTs) on individual nuts including pistachios, is now lessening these concerns^(3,47–49)^, although education of health care professionals on the health benefits of nuts remains a recognised need^(46)^.

Large epidemiological studies have reported that nut intake is inversely associated with adiposity and weight gain^(50)^ (see Table 1). The Harvard's prospective Nurses’ Health Study shows that women who consume nuts more often than twice a week gain less weight and have a lower risk of obesity than peers who seldom consume nuts^(23)^. In addition, three prospective, longitudinal cohorts among health professionals in the US (Health Professionals Follow-up Study, 1986 to 2010; Nurses’ Health Study, 1986 to 2010 and Nurses’ Health Study II, 1991 to 2011) observed that increasing intakes of nuts by 0⋅5 servings/d was associated with a lower risk of obesity^(48)^. The European Prospective Investigation into Cancer and Nutrition – Physical Activity, Nutrition, Alcohol, Cessation of Smoking, Eating Out of Home and Obesity (EPIC-PANACEA) study investigated the relationship between nut intake and changes in weight over 5 years^(47)^. This study, which included 373 293 men and women, 25–70 years old, from ten European countries in the EPIC study, showed that intake of nuts was associated with reduced weight gain and a lower risk of becoming overweight or obese. On average, study participants gained 2⋅1 kg (sd 5⋅0 kg) over 5 years. Compared to non-consumers, participants in the highest quartile of nut intake had less weight gain over 5 years and had a 5 % lower risk of becoming overweight or obese. Intake of nuts was associated with reduced weight gain and a lower risk of becoming overweight or obese. A recent systematic review and meta-analysis evaluated the prospective associations between food groups (whole grains, refined grains, vegetables, fruit, nuts, legumes, eggs, dairy, fish, red meat, processed meat and sugar-sweetened beverages), and the risk of overweight/obesity, abdominal obesity and weight gain^(51)^. In the dose–response meta-analysis, inverse associations were found for whole-grain, fruits, legumes, fish and nuts. However, evaluating the quality of the evidence with the Nutrigrade tool, investigators found that the dose–response meta-analytical findings provided very low to low quality of evidence that certain food groups have an impact on different measurements of adiposity risk. A meta-analysis of six prospective cohort studies with 420 890 participants to determine the association between nut consumption with metabolic syndrome and overweight/obesity showed that for every 1-serving/week increase in nut consumption, the risk for overweight/obesity was reduced by 3 %^(3)^.

Evidence from intervention studies is consistent with these associations. A meta-analysis of thirty-three RCTs indicates that nut-rich diets do not increase body weight, BMI or waist circumference, in comparison with various control diets^(24)^. Another meta-analysis of pooled data from sixty-two randomised feeding studies looking at the risk of metabolic syndrome and overweight/obesity concluded that nut supplementation, compared with control diets, was associated with small but significant reductions in body weight (fifty-six studies), BMI (thirty-nine studies) and waist circumference (twenty-three studies)^(3)^.

Guarneiri *et al.*^(52)^ conducted a systematic review and meta-analysis of fifty-five nut intervention trials, with and without dietary substitution instructions, to determine if there were changes in body weight and composition. In studies without dietary substitution instructions, there was no change in body weight. In studies with dietary substitution instructions, there was also no change in body weight; however, there was a significant decrease in percentage body fat. There was no change in BMI or waist circumference for either category of studies. A recent randomised, controlled, two-arm study of overweight individuals tested whether including nuts (42 g (1⋅5 oz)/d) in a weight loss and maintenance program interfered with weight management compared to a refined carbohydrate pretzel snack^(53)^. Both arms were fed hypoenergetic diets followed by an isoenergetic weight maintenance program for 12 weeks. Participants in both groups experienced significant weight loss. However, there was no difference in weight loss between those in the mixed nuts and the pretzel snack group. Those in the mixed nut group showed a significant increase in satiety at 24 weeks.

## Pistachios and body weight

Looking specifically at pistachios (Table 2), confirming these results, a recent meta-analysis and systematic review of RCTs evaluated the relationship between pistachio intake and various indicators of adiposity. This review included eleven RCTs, with a total of 1593 participants. It reported that a diet with pistachios reduced BMI and had no significant effects on body weight and waist circumference^(54)^.

Two RCTs investigating the effects of pistachios on cardiometabolic risk factors in individuals with metabolic syndrome included measures of body weight and/or anthropometry and show that adding pistachios to the diet improves dietary nutrient intake without affecting body weight^(55,56)^. Wang^(55)^ enrolled ninety participants with metabolic syndrome and provided dietary counselling according to the guidelines of the American Heart Association Step I diet. Energy intake was not restricted and all participants were instructed not to alter their usual activity and exercise. After a 4-week run-in period study participants were randomised to consume 42 g or 70 g/d pistachios or no pistachios for 12 weeks and to avoid other tree nuts. There were no changes in body weight or waist-to-hip ratio in any of the groups. In a similar RCT, sixty participants with metabolic syndrome were assigned to consume 20 % of total energy from pistachios (intervention group) *v.* standard dietary guidelines for 24 weeks. Both groups were given similar diet and exercise advice. The pistachio intervention was associated with a significant reduction in waist circumference, compared to the control (*P* < 0⋅02), despite no change in weight^(56)^.

Two additional RCTs examined the effect of pistachios in a weight loss intervention with overweight and obese individuals^(29,57)^. Li *et al.* prescribed an isoenergetic, reduced-energy diet that included an afternoon snack of either 53 g pistachios or 56 g pretzel (control group) to fifty-nine participants in a 12-week weight loss intervention^(57)^. The pistachio and pretzel snacks were included in the daily energy allowance, which was set at 500 kcal below participants’ resting metabolic rate. Significant reductions in weight (*P* < 0⋅01) and BMI (*P* < 0⋅05) were seen in both groups from baseline and by 12 weeks, participants in the pistachio group experienced a significantly greater reduction in BMI (4⋅3 *v*. 2 % of BMI) and triacylglycerols (*P* < 0⋅02) than the control group^(57)^.

Rock *et al.* assigned one hundred men and women with overweight/obesity (BMI 27–40 kg/m^2^) to a 4-month behavioural weight loss intervention. All participants were advised to reduce daily energy intake by 500–1000 kcal and increase the physical activity in order to achieve a 1–2 pound/week weight loss (control group). Half of the participants were prescribed 42 g/d of pistachios (approximately 18 % of energy intake) within their weight loss diet^(29)^. Both groups reduced BMI and waist circumference from baseline (*P* ≤ 0⋅05), although the weight lost did not reach significance for either group. Between-group differences included a lower intake of sweets (*P* ≤ 0⋅05) and a more favourable ratio of poly- and mono-unsaturated fatty acids to saturated fatty acids (*P* ≤ 0⋅05) for the pistachio group.

The effects of daily pistachio intake on body weight, anthropometry and dietary intake have also been investigated in RCTs in healthy, non-obese women^(8,58,59)^. In a randomised, cross-over study with 10-week intervention periods, inclusion of 20 % of daily energy as pistachios in the diet of forty-eight healthy, normal-weight women did not lead to changes in body weight, blood lipids or blood pressure. However, diet quality improved in the pistachio condition, including an increase in unsaturated fat and dietary fibre intake^(59)^.

## Regular intake of pistachios, body weight maintenance, appetite control and diet quality

Two RCTs were conducted in adult French women (Table 2). The French population has a relatively lower rate of overweight/obesity than most developed countries^(60)^. The National Nutritional Guidelines^(43)^ stress the importance of maintaining a healthy weight while recommending regular nut intake. In some segments of the population, particularly adult women, concerns about weight control might antagonise the intake of energy-dense foods such as nuts.

In a parallel design RCT, Fantino *et al.* investigated the effects of adding pistachios to the daily diet on body weight and composition (DXA), appetite, energy and nutrient intake in healthy French women^(8)^. Sixty healthy pre-menopausal, women were randomised to consume their habitual diet throughout a 12-week intervention and either, to add a mid-morning snack of pistachios (44 g portion of dry roasted, low salt, shelled American pistachios, approximately 250 kcal); or, for the control group, to avoid all nuts. In addition to assessing changes in body weight and composition between the beginning and the end of the intervention, additional test sessions took place under laboratory conditions before and after the intervention in order to examine acute appetite responses (changes in hunger, desire to eat and fullness in all participants), as well as any compensatory behaviour affecting spontaneous food intake following the consumption of a pistachio load (44 g)^(47)^.

**Fig. 2.**
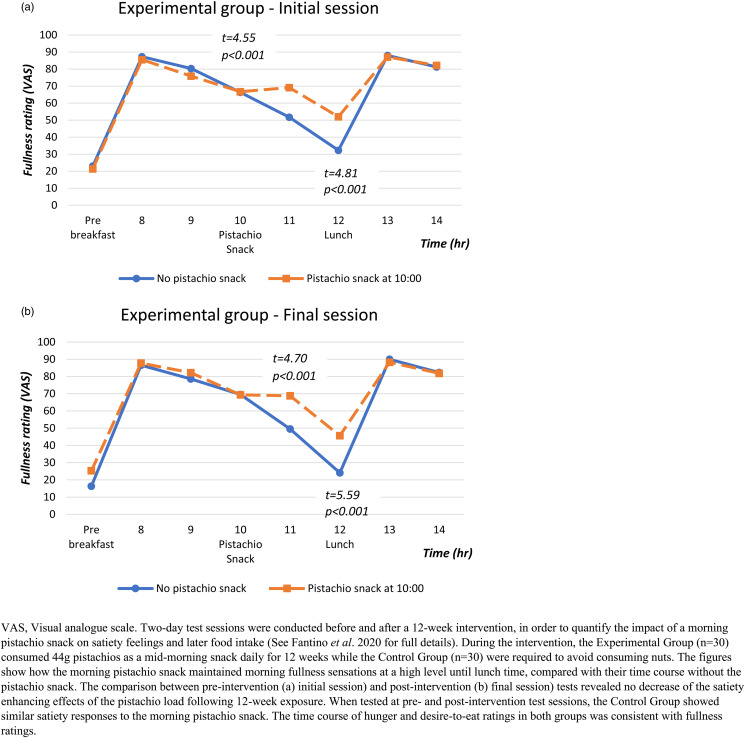
Time course of fullness ratings (visual analogue scale) with and without morning snack (44 g pistachios) under laboratory conditions^(46).^

The 12-week intervention with or without a daily morning pistachio snack did not affect body weight, lean body mass or body fat in the participants. Visual analogue scale (VAS) scores, used to measure appetite during laboratory sessions, evidenced a significant increase in fullness (Fig. 2) and a significant reduction in desire to eat and hunger until lunchtime, following consumption of the pistachio snack. In agreement with these appetite scores, spontaneous energy intake decreased at the meal following the snack (26⋅3 % of the pistachios energy (*P* < 0⋅001)), compared to the test session without the pistachio snack. A trend for a reduction in energy intake was also observed in later eating events (dinner on the same day and meals on the following day). Overall, spontaneous energy intake decreased after consuming the pistachio snack, representing 40⋅5 and 35⋅5 % of the energy provided by pistachios on the day of consumption and the following day, respectively. The combined 2-day energy intake was significantly lower (*P* < 0⋅01) in the pistachio snack session compared to the control. The lower energy intake was due to a decrease in carbohydrate intake, mainly starch and sugar, at lunchtime following the morning snack. These compensatory decreases in food intake were the same in test sessions held before and after the 12-week intervention, in both participants of the experimental and the control groups, which suggests that compensatory decreases in spontaneous intake were triggered by the immediate post-ingestive effects of pistachio consumption and were not modified by 12-weeks of daily pistachio snacks.

**Fig. 3.**
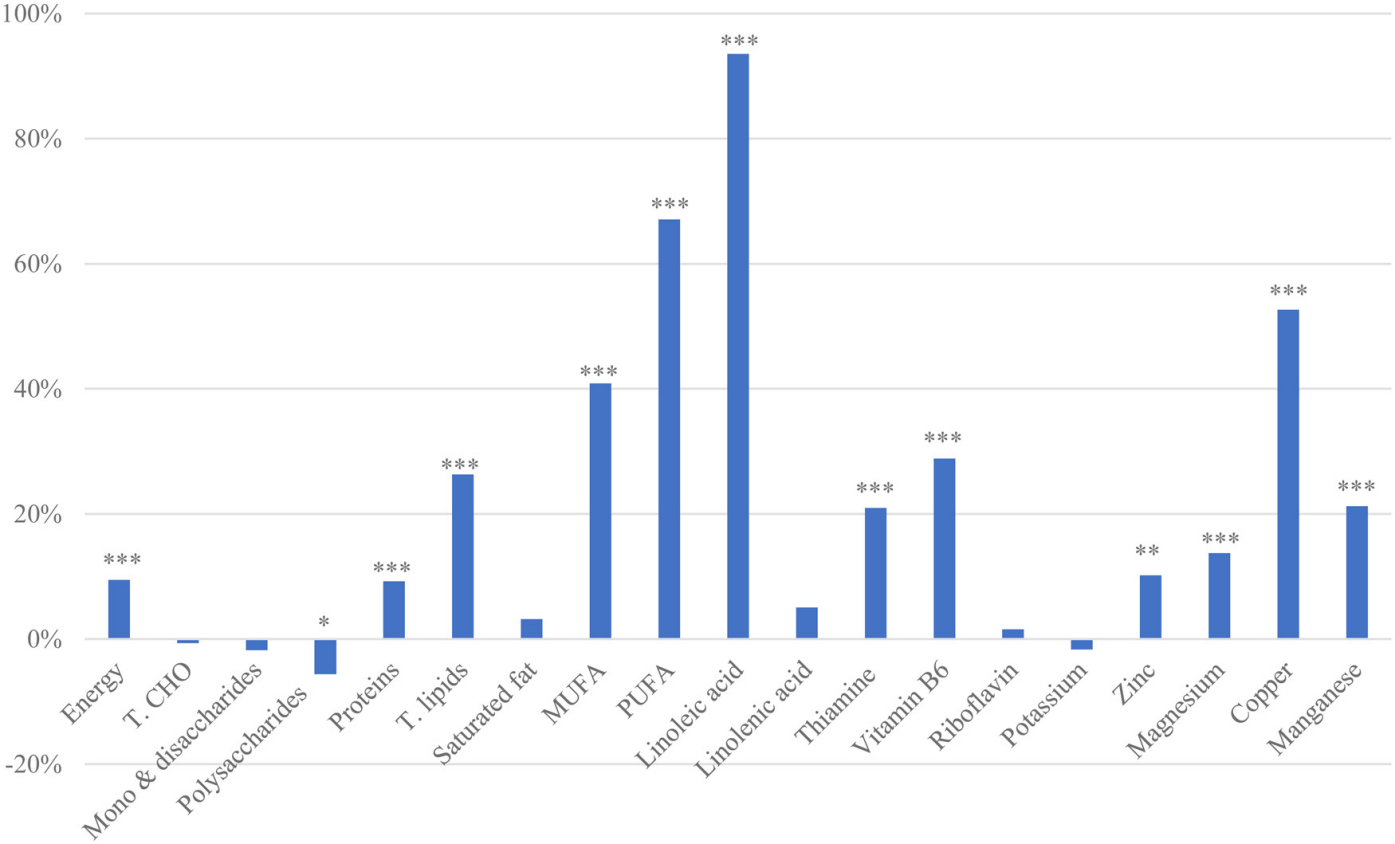
Percent differences in nutrient intake over 2 days with or without a pistachio snack (44 g)^(46)^. T.CHO, total carbohydrate; T. lipids, total lipids; MUFA, mono-unsaturated fats; PUFA, polyunsaturated fats. Two-tailed paired *t*-test: *P *<* 0⋅05, **P *<* 0⋅01, ***P *<* 0⋅001. Total percentage difference in *ad libitum* intakes in two-day sessions with pistachio snack minus identical sessions without snack. The intake data include the pistachio snacks plus the spontaneous food intake over two successive days. *n* 57 healthy women.

Importantly, despite a reduction in post-snack energy intake, diet quality improved on days with pistachio consumption, with intakes of protein, fat, MUFA, PUFA, zinc, thiamin, copper, manganese, magnesium, vitamin B6 and linoleic acid significantly higher than in sessions without a pistachio snack (*P* < 0⋅01) (Fig. 3). No changes were seen in saturated fat intake^(8)^.

This study builds on an earlier RCT in sixty healthy, normal weight (BMI 18⋅5–25 kg/m^2^) French women that explored the nutritional benefits of ingesting pistachios at the traditional mid-afternoon snack in the French diet (the ‘goûter’)^(58)^. The participants consumed either 56 g pistachios (315 kcal) or an energy- and protein-matched cheese aperitif biscuit at the afternoon ‘goûter’ as part of their normal diet for 4 weeks. Neither snack affected body weight, anthropometry or body composition but the pistachio snack was associated with a higher intake of potassium, copper, thiamin and vitamin B6 compared to the biscuit. Both snacks induced similar sensations of satiety and post-snack energy intake during the rest of the day.

The absence of weight gain in pistachio consumers in these studies, like other nut consumption studies, suggests the action of adequate compensatory responses. Several biological mechanisms can contribute to explain why nut consumption does not promote weight gain. Firstly, not all of the fat in nuts is absorbed following consumption, resulting in an overestimation of their energy contribution to the diet^(49)^. Nuts provide less metabolisable energy *in vivo* than that calculated by proximate analysis and standardised Atwater factors, as has been demonstrated in walnuts, almonds and pistachios^(61–63)^. Secondly, nuts are rich in unsaturated fats, which may have a greater post-prandial thermogenic effect than saturated fats, resulting in less fat storage^(64)^. Finally, nuts seem to enhance satiety processes, as evidenced by changes in appetite sensations following consumption and decreased intake at the subsequent eating occasions^(8,49,58)^. Nuts are high in fibre and protein, which promote satiety. In addition, nuts require considerable chewing. The time of oral processing (the oral activity needed to process food) is a factor contributing to enhance satiety^(65)^.

An additional relevant consideration highlights a unique characteristic innate to in-shell nuts. Previous studies^(66,67)^ have shown that participants consumed fewer in-shell compared to shelled pistachios, over a set time period. The authors concluded that this was due either to the additional time needed to shell the pistachios or the visual cue of the extra volume perceived when the pistachio shells are left in sight^(66)^. Thus, when consuming pistachio nuts, which are usually purchased in-shell, the act of shelling the nuts helps limit over consumption.

## Glycaemic control beyond energies: a lesson from pistachios

Research suggests that regular, moderate intakes of nuts, including pistachios are beneficial for blood glucose control in addition to improving parameters of blood lipid metabolism in both healthy adults and those with metabolic diseases^(3,68,69)^. Three experimental scenarios have demonstrated beneficial effects for pistachios on glycaemic control. When consumed alone, they have minimal effects on blood glucose^(70)^; when added to high-carbohydrate foods, pistachios attenuate post-prandial glucose levels in healthy participants and participants with metabolic diseases^(70)^; in persons with metabolic syndrome, daily consumption of pistachios attenuates post-prandial glycaemia, increases glucagon-like peptide levels and may have insulin-sparing properties (reducing the amount of insulin required to achieve a given level of glycaemic control)^(71,72)^. Pistachios’ composition includes several candidates that can explain these findings, aside from their low carbohydrate content, including their fatty acid profile, fibre, anti-inflammatory compounds and antioxidants content^(73)^.

Maintaining healthy blood glucose control during pregnancy is important for normal short- and longer-term pregnancy outcomes. Practical dietary guidance is warranted at this time when women are responsive to life-style advice. Gestational diabetes mellitus (GDM) is diabetes diagnosed for the first time during pregnancy^(74)^. This general term includes both GIGT (abnormal 50 g glucose tolerance test) and frank GDM (fasting glucose ≥5⋅1 mmol/l, 1 h glucose ≥10 mmol/l or 2 h glucose ≥8⋅5 mmol/l during a 2 h 75 g oral glucose tolerance test (OGTT) for first and subsequent trimester 24–28 weeks)^(75)^. Worldwide estimated GDM prevalence is 9⋅8–25⋅5 %^(9)^ and has a significant impact on the health of both mother and baby^(76)^.

GDM is an independent risk factor for preeclampsia^(77)^, increases the risk of preterm birth^(78)^ and the likelihood of birth by caesarean section^(79)^. GDM is associated with a 3× higher rate of foetal macrosomia^(80)^, with second and third trimester post-prandial blood sugar measurements highly correlated with foetal birth weights. For the infant, macrosomia increases the risk of shoulder dystocia, clavicle fractures, brachial plexus injury and neurological impairments and increases the rate of admissions to the neonatal intensive care unit. For the mother, macrosomia increases the risk for delivery complications. Macrosomia in GDM is mainly due to the increased insulin resistance of the mother. Foetal macrosomia increases the risk of becoming overweight or obese during adolescence and to develop type II diabetes (T2DM) later in life^(81)^. A third of women who develop GDM will go onto develop T2DM in later life and at least half will develop GDM during a second pregnancy.

Effective treatment for gestational diabetes includes diet, exercise and insulin therapy^(82)^. The choice of healthy food is a key component. Tryggvadottir *et al.* observed that adhering to a healthy prudent diet which included nuts and seeds, seafood, fruits and vegetables was associated with reduced risk for GDM and GDM-associated complications compared to Westernised diets, especially among women with overweight or obesity^(83)^. Ruiz-Garcia *et al.* assessed GDM risk factors in 1750 pregnant women from the St. Carlos Gestational Study between 24 and 28 weeks of gestational age (GA). A semiquantitative Food Frequency Questionnaire was used to evaluate diet during pregnancy. They observed that adherence to a low-risk nutritional pattern, from early pregnancy on, could be an effective strategy for GDM prevention^(84)^. The same group showed that high adherence to consumption of six predefined food targets of the Mediterranean diet (fruits and vegetables, nuts, extra virgin olive oil) was associated with a lower risk of GDM^(85)^. This has been replicated by the ESTEEM multicentre randomised trial in the UK^(86)^. Similarly, Asadi *et al.* showed in a case–control study at six healthcare centres in Iran that pre-pregnancy adherence to the prudent dietary pattern was significantly associated with reduced risk of GDM^(87)^. The prudent dietary pattern was associated with ‘high intakes of fruits, low-fat dairy, potato, egg, fish, poultry, nuts, organs meat and red meat’. A systematic review of intervention and observational studies on T2DM outcomes in women with a history of GDM showed better outcomes among those consuming diets rich in fruit, vegetables, nuts, fish and legumes, and low in red and processed meats and sugar-sweetened beverages^(88)^. Data from the Australian Longitudinal Study on Women's Health (2003–2012) which included 3853 women without pre-existing diabetes showed that a Mediterranean style dietary pattern was associated with lower GDM risk^(89)^. Data collected from the longitudinal Nurses’ Health Study II (1991–2001) indicate that pre-pregnancy intake of animal protein, in particular red meat, is significantly and positively associated with GDM risk, whereas consumption of vegetable protein, specifically nuts, is inversely associated with GDM risk. Moreover, their findings suggest that among women of reproductive age, the substitution of vegetable protein for animal protein, as well as substitution of some healthy protein sources (e.g., nuts, legumes, poultry and fish) for red meat may potentially lower GDM risk^(90)^.

Interest in the specific effects of pistachios in GDM stems from results of RCTs^(28,56)^ and prospective studies that suggest regular pistachio consumption can lower blood glucose levels^(91)^. A recent study has investigated the effects of consuming pistachio nuts on GDM (Table 3). Assaf-Balut *et al.* conducted a prospective, RCT (St. Carlos Gestational Diabetes Mellitus Prevention Study) to evaluate the incidence of GDM with either a control diet (standard Mediterranean diet with limited fat intake) or a Mediterranean diet supplemented daily with at least 40 ml extra virgin olive oil (EVOO) and a handful (25–30 g) pistachios. One thousand normoglycemic (<5⋅1 mmol/l) pregnant women at 8–12th gestational weeks (GW) were recruited for the study to assess the effect of the intervention on GDM incidence at 24–28 GW. A total of 874 women completed the study. The supplemented Mediterranean diet reduced the incidence of GDM rate from 23⋅4 % (in the control group) to 17⋅1 % and improved maternal and neonatal outcomes (significantly lower rates of insulin-treated GDM, prematurity, emergency caesarean sections, perineal trauma and small and large for GA new-borns)^(92)^ (Table 5). Health benefits to the infant persisted during the first two years of life: the offspring of mothers receiving the supplemented Mediterranean diet during pregnancy had fewer hospital admissions requiring antibiotic and corticosteroid treatment, and fewer admissions related to asthma/bronchiolitis, especially in women with pre-gestational BMI <25 kg/m^2(93)^. A later sub-analysis of the data collected in the St. Carlos Gestational Diabetes Mellitus Prevention Study showed that mothers receiving the pistachio and EVOO-enriched diet had over 50 % lower risk of composite adverse maternofoetal outcomes, including lower mean fasting glucose and HbA levels, at 24–28th and 36–38th GW, in the intervention group compared to the standard care control group^(94)^. The authors conclude from this study that a MedDiet, enhanced with EVOO and nuts, such as pistachios, ‘might be a potentially adequate diet for pregnant women’ and ‘current recommendations to limit fat consumption during pregnancy need to be revised’^(92)^.

**Table 5.** Maternal pregnancy and neonatal outcomes following daily supplementation of extra virgin olive oil and pistachios

	All women (normo-glycaemic (<5⋅1 mmol/l) at 8–12 GW^(92)^		Normo-glycaemic throughout pregnancy^(94)^	
	Control group (*n* 440)	Intervention group (*n* 434)	Control group (*n* 337)	Intervention group (*n* 360)
Maternal outcome				
GDM	103 (23⋅4 %)	74 (17⋅1 %)*	0	0
Treatment of GDM				
Nutrition	70 (65⋅4 %)	60 (81 %)	0	0
Insulin	33 (32 %)	14 (19 %)*	0	0
Delivery				
Emergency section	31 (51⋅7 %)	9 (15 %)***	25 (7⋅4 %)	8 (2⋅2 %)**
Perineal trauma	48 (10⋅9 %)	14 (3⋅2 %)***	49 (14⋅5 %)	13 (3⋅6 %)***
Neonatal outcomes				
Gestational age at birth (weeks)	39⋅6 ± 1⋅4	39⋅6 ± 1⋅2	39⋅6 ± 1⋅3	39⋅7 ± 1⋅3
Prematurity (<37 GW)	17 (3⋅8 %)	5 (1⋅2 %)**	11 (3⋅6 %)	4 (1⋅1 %)
Birthweight (g)	3215 ± 480	3250 ± 391	3219 ± 465	3269 ± 396
Small for GA (<10 percentile)	25 (5⋅7 %)	5 (1⋅2 %)***	14 (4⋅2 %)	4 (1⋅1 %)*
Large for GA (<10 percentile)	18 (4⋅1 %)	4 (0⋅9 %)**	11 (3⋅3 %)	3 (0⋅8 %)*

GW, gestational weeks; GDM, gestational diabetes mellitus; GA, gestational age.

Control diet: standard Mediterranean diet with limited fat intake; Intervention; Mediterranean diet supplemented daily with >40 ml extra virgin olive oil and handful (25–30 g) pistachios.

*P *<* 0⋅05, **P < 0⋅01, ***P ≤ 0⋅001.

A study conducted in Chinese women^(9)^ investigated the specific role of pistachios in GDM using a randomised, controlled, cross-over design to assess the acute effect of pistachio intake on post-prandial glucose, insulin and gut-derived incretin hormones (GLP-1, GIP) in comparison to an isoenergetic test meal of WWB. Both nuts and WWB are included on the standard recommended healthy snack lists for diabetics in China as whole grains can help reduce or retard blood glucose response^(9)^; and low-fat diets are recommended in China to help improve blood glucose levels in patients with T2DM^(95)^.

Seventy-three women, 23–39 years, were recruited from the prenatal care clinic in the Shanghai Jiaotong University Affiliated Sixth People's Hospital (Shanghai, China) at the routine 24–28th GW GDM screening visit^(9)^. Primipara women from one unique ethnic group (the ‘Han’) testing positive from a standard 50 g OGTT were eligible to participate and were assigned to separate groups, either GDM or GIGT, following a second overnight fast and diagnostic 75 g OGTT. Women who fulfilled at least one of the following criteria were diagnosed GDM: fasting glucose ≥5⋅1 mmol/l, 1 h glucose ≥10 mmol/l or 2 h glucose ≥8⋅5 mmol/l during the 2 h 75 g OGTT. The diagnosis of GIGT was made when participants tested positive with 50 g glucose tolerance test but did not meet the criteria of GDM. Thirty women with GIGT and twenty-nine with GDM completed the study. In a cross-over design, all participants were further randomised to receive either 42 g pistachios or 100 g (2 slices) WWB on study day 1; followed by a 1-week washout and the alternate snack on study day 2. The foods were matched for energies and were similar for fibre and protein, while carbohydrate and fat contents differed widely for the WWB and pistachio conditions (Table 6). Test days followed standard OGTT methods. Fasting blood samples were collected to determine baseline levels of glucose, insulin and incretins. Women were then given either WWB or pistachios and consumed the load within 15 min. Blood samples were collected 30, 60, 90 and 120 min after ingestion.

**Table 6.** Nutrients (per serving size) provided by isoenergetic test meals of whole wheat bread and pistachios^(9)^

	Whole wheat bread	Pistachios
Serving size (g)	100 (2 slices)	42 (1⋅5 oz)
Energy (kJ/kcal)	1004/240	979/234
Fat (g)	4⋅1	18⋅9
Carbohydrate (g)	41⋅9	12⋅18
Fibre (g)	5	4⋅2
Protein (g)	10⋅3	8⋅82

**Fig. 4.**
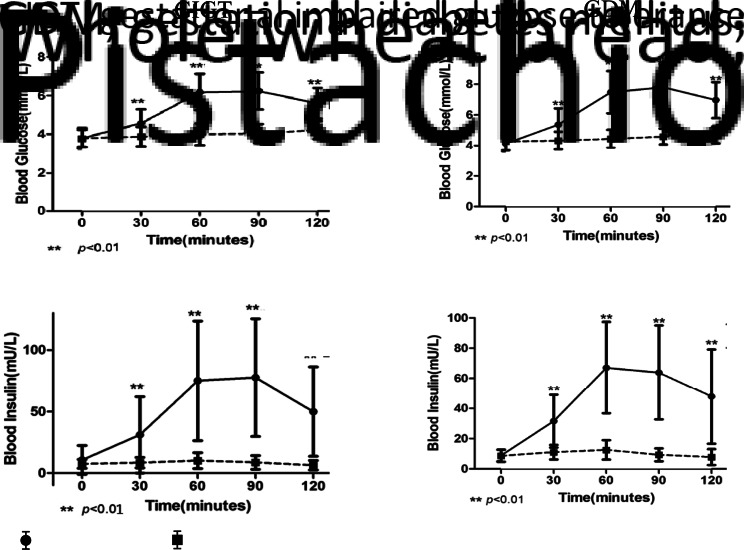
Effect of whole wheat bread and pistachios on glucose and insulin levels in women with GIGT or GMD^(23)^.

Pistachio consumption did not increase the blood glucose and insulin levels compared to baseline in both GIGT and GDM groups. Conversely, WWB consumption significantly increased blood glucose and insulin levels. Compared to pistachio consumption, blood glucose and insulin levels at 30, 60, 90 and 120 min post-prandial (Fig. 4), as well as AUC_glucose0−120 min_ and AUC_insulin0−120 min_, were significantly higher after WWB consumption in both GIGT and GDM participants. This was not unexpected: the difference in carbohydrate content between pistachios and WWB explains differences in post-prandial glucose and insulin responses between pistachio and WWB intake.

Glucagon-like peptide-1 (GLP-1) and glucose-dependent insulinotropic polypeptide (or gastric inhibitory polypeptide, GIP) are the primary incretin hormones secreted from the intestine following glucose/food consumption, to stimulate insulin secretion and hence reduce post-prandial glucose levels. This potentiation of insulin secretion by gut hormones after an oral glucose load is crucial for controlling post-prandial glucose excursions. Other impacts include slowing down the gastric emptying time. GLP-1 is a key hormone responsible for satiety. Impaired post-prandial GLP-1 response was previously reported in pregnant women with GDM. In both GIGT and GDM groups, Feng *et al.* observed significantly higher GLP-1 levels at 90 and 120 min after pistachio compared to WWB intake. Also, significant lower GIP levels were seen at 30 and 60 min in GDM patients or 120 min in GIGT patients after pistachio compared to WWB intake (Table 7).

**Table 7.** Effect of whole wheat bread and pistachios on incretin (GIP and GLP-1) levels in women with GDM or GIGT^(9)^

	GDM				GIGT			
	GIP (pmol/l)		GLP-1 (pmol/l)		GIP (pmol/l)		GLP-1 (pmol/l)	
Time (minutes)^a^	WWB	P	WWB	P	WWB	P	WWB	P
0	6⋅75 ± 0⋅78	6⋅63 ± 0⋅74	4⋅46 ± 0⋅50	4⋅69 ± 0⋅49	15⋅33 ± 3⋅98	13⋅91 ± 4⋅50	3⋅50 ± 0⋅79	3⋅67 ± 0⋅75
30	35⋅91 ± 22⋅43	23⋅26 ± 22⋅26*	6⋅53 ± 4⋅24	5⋅60 ± 2⋅63	43⋅93 ± 47⋅82	29⋅15 ± 42⋅04	3⋅59 ± 3⋅18	3⋅49 ± 3⋅44
60	63⋅92 ± 32⋅10	44⋅93 ± 27⋅89*	7⋅33 ± 3⋅15	7⋅08 ± 2⋅77	72⋅25 ± 49⋅63	53⋅01 ± 55⋅67	3⋅30 ± 2⋅67	5⋅07 ± 4⋅66*
90	57⋅52 ± 25⋅06	46⋅82 ± 31⋅35	4⋅87 ± 3⋅01	7⋅45 ± 3⋅51*	67⋅57 ± 53⋅51	50⋅65 ± 46⋅59	2⋅88 ± 2⋅37	5⋅53 ± 4⋅75*
120	59⋅53 ± 28⋅35	50⋅29 ± 33⋅65	4⋅37 ± 3⋅12	6⋅85 ± 3⋅65*	66⋅62 ± 43⋅60	52⋅99 ± 30⋅94*	2⋅61 ± 3⋅11	4⋅22 ± 4⋅25*

GIP, gastric inhibitory polypeptide; GLP-1, glucagon-like peptide-1; GDM, gestational diabetes mellitus; GIGT, gestational impaired glucose tolerance; WWB, whole wheat bread; P, pistachios.

a Time after consumption of the meal.

*P < 0⋅05 between treatments.

Results from this study in Chinese pregnant women are indicative that for those with GDM and GIGT, pistachios provide an effective alternative to the usual low-fat, high-carbohydrate whole-grain food (WMB) recommended to improve post-prandial glucose, insulin, GIP and GLP-1 response. These studies provide evidence that pistachio nuts, with their healthy nutrient profile, can be an ideal choice for healthy snacks during pregnancy.

## Recommendations for future research with pistachios

The general consensus from epidemiological studies is that usual consumers of nuts have a lower BMI *v.* non-consumers and results from experimental studies of nut supplementation broadly show no increase in body weight, despite nuts’ energy density. Several plausible explanations for this lack of adverse effects on body weight are discussed earlier, including energy compensation in subsequent eating occasions. Fantino *et al.* observed about 40 % of the pistachio snack energy being compensated for over two days following consumption of a pistachio load^(8)^. Further research is needed to identify the full compensatory mechanisms contributing to the lack of adverse effects on body weight or composition in pistachio consumers. Identifying these mechanisms will be important in order to understand how pistachios can beneficially contribute to healthy nutrition and weight management.

Research indicates that nut consumers have better nutrient adequacy and diet quality than non-nut consumers^(25,26)^. Given the now well-established beneficial effects of including nut snacks in weight management interventions, over set time periods, it would be informative to examine how long lasting, nut snacking behaviour changes can be sustained and to assess consequent, longer-term health benefits. Although it is challenging to undertake well-controlled dietary studies for more than 12 weeks, longer-term observations of the effects of pistachios on weight management are needed to establish strong nutritional benefits, to further validate present findings and to offer opportunities to test potential mechanisms.

Studies in other populations are needed to extend the findings obtained in healthy, non-obese, French women^(8,58)^, men, other age groups and people with obesity, diabetes, metabolic syndrome or other nutrition-related diseases. The optimal time for consuming pistachios in order to maximise the decrease in energy intake at the next meals should be investigated. Quality of life effects, including sleep patterns during pistachio snacking studies, may also prove interesting, given that pistachios contain melatonin^(96)^.

The significant amounts of lutein and zeaxanthin in pistachios^(12)^ (1160 μg/100 g)^(10)^ open a novel and compelling area of research, as these carotenoids are taken up and concentrated in the central area of the retina, the macula, where they may have a role in protecting the tissue from phototoxic damage^(97)^. This may be important in age-related macular degeneration, a major cause of visual impairment^(98,99)^. Lutein is also the predominant carotenoid in human brain tissue. Johnson^(99)^ found a positive correlation between the amount of lutein in the retina and measures of cognitive function. The relationship between lutein and zeaxanthin and visual and cognitive health throughout the lifespan warrants further investigation.

Pregnancy is a known time for optimal dietary influence, where a woman is concerned not only for her own health but that of her developing offspring. Research with pistachios suggests that dietary quality as regards fat and carbohydrate energies has the potential to beneficially impact GDM^(9)^ and therefore specific practical dietary advice for expectant mothers, in particular, those at risk of developing GDM, needs further consideration. Further research is warranted on the impact on weight management, glycaemic control and wider whole diet quality and health effects for both mother and baby, of consuming a natural, nutrient-dense food such as pistachios throughout the entire pregnancy. It would be worthwhile to explore the potential for pistachio snacking to reduce the risk of progressing from impaired glucose tolerance to full gestational diabetes. Potential benefits could also be considered in women with the polycystic ovary syndrome (PCOS), given the links between PCOS, obesity and poor fertility^(100)^.

Worldwide prevalence of obesity has nearly tripled in recent years (1975–2016)^(101)^. The combined health benefits from active lifestyles and a healthy diet are advocated to reduce the obesity burden in the entire population^(102)^. It is also well established, however, that changing life-style habits (both dietary and physical activity/exercise) is difficult and the need for effective strategies has never been more apparent. Weight management is also crucial for optimal sports performance and many motivated athletes struggle with long-term dietary regimes. Although beyond the scope of the present paper, a case study presented at FENS^(103,104)^ provides a clue to potential research opportunities for finding such strategies. In this study the weight and performance of one athlete were examined following a three meal-a-day healthy vegan diet including 100 g pistachio snacks. After 5 months on this diet, the participant was still adhering to the pistachio snacks and had improved his body composition, reducing weight and increasing muscle mass. This illustrates the benefits of long-term, sustainable approaches to weight management, whether for athletes or more sedentary consumers. This is an area worthy of further enquiry.

## Conclusions

Pistachio nuts are nutrient dense, high in fibre and good quality plant protein, providing a wide array of micronutrients and health-protective bioactive compounds. Pistachios are a physiologically beneficial, tasty and versatile alternative to many foods, particularly unhealthy snacks. Their practical advantages could benefit different population groups, contributing to a healthy balanced, more plant-based diet. The present paper has reviewed the research about weight management and gestational diabetes and provided research recommendations to extend our understanding of the wider potential role that nuts such as pistachios could play in improved diet quality and health. Given the global necessity to address both sustainability and nutrition/health needs in a complementary manner, pistachios appear a promising candidate for healthy 21st-century nutrition and sustainable agricultural practices.
